# Education Research: Validity and Reliability of the Neurophobia-Combined Measure (NCM) in Irish Medical Students

**DOI:** 10.1212/NE9.0000000000200291

**Published:** 2026-03-17

**Authors:** Liah McElligott, Caitriona Cahir, Diane M. Gillan, Norman Delanty, Susan Byrne, Noel Gerry McElvaney, Eavan McGovern

**Affiliations:** 1 School of Postgraduate Studies, Royal College of Surgeons in Ireland, Dublin, Ireland;; 2 Department of Neurology, Beaumont Hospital, Dublin, Ireland;; 3 Data Science Centre, School of Population Health, Royal College of Surgeons in Ireland, Dublin;; 4 Department of Neuropsychology, Beaumont Hospital, Dublin, Ireland;; 5 School of Medicine, Royal College of Surgeons in Ireland, Dublin;; 6 Future Neuro SFI, Research Centre for Neurological Disease, RCSI, Dublin, Ireland; and; 7 Children's Health Ireland, Crumlin Hospital, Dublin, Ireland.

## Abstract

**Background and Objectives:**

Neurophobia, defined as a fear of neurology and the neurosciences, is a recognized barrier in medical education and clinical practice. It affects one-third of medical students internationally, yet measurement approaches remain inconsistent. This study aimed to validate the Neurophobia-Combined Measure (NCM), a psychometric tool for assessing neurophobia in medical students.

**Methods:**

NCM items were developed from literature, expert input, and existing measures of interest, confidence, and anxiety. Final-year medical students at the Royal College of Surgeons in Ireland completed the NCM alongside the Test Anxiety Inventory (TAI-5), State-Trait Anxiety Inventory (STAIT-5), and a summative Multiple Choice Questionnaire Examination (MCQE). Exploratory and confirmatory factor analyses were conducted to evaluate dimensionality. Model fit was assessed using structural equation modeling indices. Internal consistency was estimated with Cronbach alpha and McDonald omega. Criterion, convergent and discriminant validity were assessed via correlations with MCQE, TAI-5, and STAIT-5. Receiver operating characteristic (ROC) analysis tested diagnostic accuracy, and invariance was explored across sex and academic entry status.

**Results:**

A total of 311 students (mean age = 24 years, SD = 3; 64% female; 70% undergraduate-entry) completed all measures. Exploratory factor analysis supported a 2-factor structure reflecting (1) perceived neurology difficulty/complexity and (2) confidence/interest. The 2-factor model demonstrated good fit, χ^2^ (26) = 75.77, *p* < 0.001, Root Mean Square Error of Approximation = 0.079, 90% CI [0.058, 0.099], CFI = 0.93, Tucker-Lewis Index = 0.90, and Standardized Root Mean Square Residual = 0.053. Internal consistency was acceptable (Cronbach α = 0.80; McDonald ω = 0.81). Item Response Theory model comparisons further supported the 2-factor structure, with separate NeuroQ and Schön models showing better fit than a combined unidimensional model. Receiver operating characteristic analysis indicated strong accuracy (area under the ROC curve = 0.96). Overall, 60% of students were classified as neurophobic, with higher prevalence among female (69%) and undergraduate-entry (65%) students. Neurophobia correlated positively with trait anxiety (ρ = 0.16, *p* = 0.004, 95% CI [0.06–0.27]) and test anxiety (ρ = 0.11, *p* = 0.047, 95% CI [0.00–0.23]). Both neurophobia (ρ = −0.12, *p* = 0.041, 95% CI [–0.23 to 0.00]) and test anxiety (ρ = −0.19, *p* = 0.001, 95% CI [–0.30 to −0.08]) were negatively associated with MCQE performance.

**Discussion:**

The NCM demonstrates good reliability and validity and reflects 2 related but distinct dimensions of neurophobia. Future research should examine its applicability across institutions and educational levels.

## Introduction

Neurologic disorders represent a leading cause of disability and mortality worldwide and constitute a growing global health burden.^1^ Despite this, neurology remains both underresourced within health care systems and underrepresented in medical curricula.^2-4^ The resulting educational gap has been linked to *neurophobia*, defined as “the fear of neurology and clinical neuroscience,”^5^ which challenges the development of neurologic expertise and workforce capacity.^5-8^ Reported barriers to effective neurology education include the perceived complexity of neuroanatomy, limited clinical exposure, difficulty mastering the neurologic examination, and suboptimal teaching methods.^5,9-14^

Neurophobia is widely recognized as a multidimensional construct encompassing perceived subject difficulty, low confidence, and reduced interest in neurology.^5,6,10,13,15^ Measurement remains inconsistent across studies, leading to challenges in comparing findings and synthesizing evidence.^6,16,17^ Some studies apply broad conceptual definitions,^5,16,18^ whereas others use psychometric tools, both validated^17^ and nonvalidated,^19-21^ to operationalize the phenomenon. Continuous refinement of these scales develops accurate construct representation and psychometric rigour.^22^

Two principal instruments have been developed for this purpose, the Schön Questionnaire and the NeuroQ. The Schön Questionnaire was the earliest and most widely applied tool, assessing attitudes, perceived difficulty, lack of confidence, knowledge, and interest in neurology relative to other specialties.^19^ It captures affective and attitudinal components of neurophobia but lacks sensitivity to educational change and psychometric validation.^23-25^ Subsequently, the NeuroQ was developed as a concise, unidimensional screen designed to quantify neurophobia severity and responsiveness to teaching interventions^17^ through measuring *self-efficacy* and *perceived competence* within neurology. This tool demonstrated sensitivity to change following a structured educational program and was validated in a single preclinical cohort. The constructs measured are limited to cognitive self-assessment, omitting the affective drivers of neurophobia such as fear or disinterest.^26,27^

From a psychometric perspective, these instruments assess related yet distinct latent constructs representing correlated but separable domains within the broader framework of neurophobia. Best practice in educational measurement recommends comprehensive construct representation through multimethod assessment and structural validation.^28-30^Combing these tools, therefore, offers both conceptual and empirical advantages, enabling examination of dimensionality, discriminant validity, and higher order structure within a unified model.

To address this gap, the Neurophobia-Combined Measure (NCM) was developed to synthesize the strengths of both instruments into a concise, psychometrically robust scale encompassing attitudes, confidence, and perceived competence. The NCM aims to provide a more complete and standardized foundation for neurophobia measurement, facilitating both educational evaluation and cross-study comparability. The primary objective of this study was to develop and validate the NCM and to evaluate its reliability and construct validity. Secondary objectives included exploring associations between neurophobia, academic performance, and academic anxiety.

## Methods

### Design

**Figure 1:**
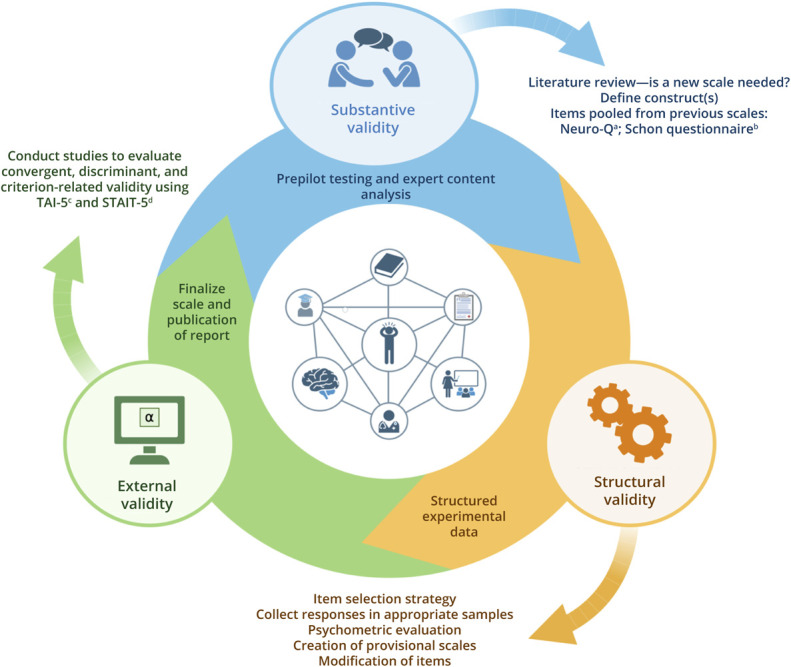
Study Design Using COSMIN Guidelines Figure created using BioRender by the corresponding author. COSMIN = the Consensus-Based Standards for the Selection of Health Measurement Instruments.

We conducted a single-center study in the School of Medicine, at the Royal College of Surgeons in Ireland (RCSI). This study employed a cross-sectional design following established guidelines for psychometric validation, including assessments of reliability, validity, and item performance.^31^ Figure 1 outlines an evidence-based approach for scale development using consensus-based standards for the selection of health measurement instruments guidelines.^32^

### Procedure

Royal College of Surgeons (RCSI) final-year medical students were recruited for this study. The study was conducted from September 2021 to December 2024. Ethical approval was obtained from the institutional Research and Ethics Committee of the RCSI (202209004). Participants were invited to complete the scale via a secure survey platform (SurveyMonkey). Data collection was pseudo-anonymized. Informed consent was obtained from all participants before data collection.

#### Participants

A total of 698 final-year medical students from RCSI Dublin were recruited over 2 academic years (2022–2023: n = 346; 2023–2024: n = 352). Data were collected from consecutive cohorts of final-year medical students to increase sample size, improve the precision of estimates, and allow assessment across more than a single cohort. Questionnaires were distributed between September and October each year. Consecutive cohorts were included to increase precision and generalizability. Eligibility required enrollment in the final medical program and informed consent; those previously involved in related studies were excluded. Participants were selected by convenience sampling, and demographics (age, sex, and entry pathway) were recorded. In the Republic of Ireland, students can enter medical school through either the undergraduate or graduate entry pathway. Undergraduate entry is typically for students who complete secondary education and meet the necessary academic and standardized testing requirements. Graduate entry is designed for students who have already completed a bachelor's degree in another field. Demographics, such as sex, was collected based on self-identified or reported sex, as stated by participants during data collection.

### Scale Development

#### The NCM

##### Item Development—Domain Identification

This scale focused on measuring neurophobia.^10,33^ To define the domain, we performed a comprehensive literature review. Neurophobia was considered well defined with the working knowledge available in the literature.^5,6,12,14,20,23,24,33-38^ Previously validated neurophobia scales were also identified at this stage. Both the NeuroQ^17^ and the Schön Questionnaire^19^ were used in scale development.

The resulting instrument, the Neurophobia-Combined Measure (NCM), is a 9-item instrument designed to measure neurophobia. Items were chosen through expert input from consultant neurologists, neuropsychologists, and academic staff. Each item is rated on a 5-point Likert scale, with higher scores indicating greater levels of neurophobia. All items used 5-point Likert scales reflecting increasing agreement. Response anchors were adapted for item context (e.g., confidence, competence) while maintaining ordinal consistency.^39-41^ For analysis, items were treated consistently as ordinal indicators. The NCM is presented in Table 1.

**Table 1:** Participant Characteristics at Baseline (n = 311)

Variable	Baseline	St err	CI (95%)	Shapiro-Wilk (*p* value)
Age (y), mean (SD)	24 (3)	0.19	24.26649–25.01325	0.00000
Female, n (%)	198 (63.7%)	—	—	0.00000
Male n (%)	107 (34.4%)	—	—	
Nonbinary n (%)	6 (1.9%)	—	—	
Undergraduate entry, n (%)	219 (70.4%)	—	—	0.07683
Graduate entry, n (%)	92 (29.6)	—	—	
*NCM*, mean (SD)	29.64 (5.04)	0.28	29.08323–30.20938	0.90176
*MCQE*, mean (SD)	10.96 (2.99)	0.17	10.63314–11.30255	0.00710
*STAIT-5*, mean (SD)	12.55 (3.89)	0.22	12.11838–12.98772	0.03108
*TAI-5*, mean (SD)	13.13 (4.06)	0.23	12.68159–13.5885	0.00350

Abbreviations: MCQE = Multiple Choice Questionnaire Examination; NCM = Neurophobia-Combined Measure; STAIT-5 = State-Trait Anxiety Inventory-5; TAI-5 = Test Anxiety Inventory-5.

##### Item Numbers

For item generation, approximately twice as many items as anticipated for the final scale were developed to ensure comprehensive coverage of the construct domains.^42^ Item wording was tailored to the target population of medical students, and reverse-scored items were included to minimize response bias.^43,44^

##### Concept Development and Reduction of Dimensionality

A conceptual clustering approach (items are grouped based on factors they have in common) was used to minimize the underrepresentation of fear and anxiety.

#### Test Anxiety and State-Trait Anxiety

Two validated short-form psychological instruments, The Test-Anxiety Inventory (TAI-5)^45,46^ and State-Trait Anxiety Inventory (STAIT-5),^47^ were administered during the same data collection period to assess test and trait anxiety.

#### Knowledge Performance

Academic performance was measured using a 17-item neurology multiple-choice examination (MCQE) assessing core final-year concepts. Items were developed by expert panel review, retaining those correctly answered by over 50% of students. Two pilot studies refined the MCQE, and questions rated as poor quality by at least 2 independent reviewers were removed (Table 2).

**Table 2:** Descriptive Statistics and Reliability of the Neurophobia-Combined Measure, Test Anxiety Inventory-5, and State-Trait Anxiety Inventory-5

Scale/item	Original code	Item statement	M	SD	Item-total *r*	α (if item deleted)
Neurophobia-combined measure (NCM)						
NCM1	SCHON1	Interest in neurology	3.52	1.14	0.58	0.79
NCM2	SCHON2	Knowledge in neurology	3.49	0.84	0.58	0.78
NCM3	SCHON3	Neurology easy/difficult	3.81	0.72	0.63	0.77
NCM4	SCHON4	Preparedness for exam	3.12	0.87	0.49	0.79
NCM5	NQ1	Difficult to understand	3.49	0.90	0.75	0.75
NCM6	NQ2	Confidence in knowledge (R)	2.60	0.93	0.67	0.77
NCM7	NQ3	Neurology more complicated	3.68	0.90	0.62	0.77
NCM8	NQ4	Confidence in studying (R)	2.50	0.84	0.69	0.76
NCM9	NQ5	Apply knowledge clinically	3.47	0.98	0.57	0.79
NCM (9 items)	—	—	29.67	5.04	—	α = 0.80
Test anxiety inventory (TAI-5)						
TAI1	—	During tests, I feel anxious	2.8	0.9	0.80	0.78
TAI2	—	I worry about failing exams	2.8	1.0	0.85	0.77
TAI3	—	My mind goes blank during exams	2.4	0.9	0.82	0.78
TAI4	—	I feel tense while studying for tests	2.7	1.0	0.87	0.77
TAI5	—	I feel calm during tests (R)	2.4	1.0	0.79	0.78
TAI-5 (5 items)	—	—	13.14	4.06	—	α = 0.81
State-trait anxiety inventory (STAIT-5)						
STAIT1	—	I feel calm	2.3	0.9	0.76	0.78
STAIT2	—	I feel tense	2.5	1.0	0.84	0.76
STAIT3	—	I feel upset	2.6	1.0	0.79	0.77
STAIT4	—	I feel relaxed	2.6	0.9	0.77	0.78
STAIT5	—	I feel worried	2.5	1.0	0.83	0.76
STAIT-5 (5 items)	—	—	12.55	3.90	—	α = 0.81

Abbreviations: NCM = Neurophobia-Combined Measure; M = mean; (R) = reverse-scored; *r* = item-total correlation; STAIT-5 = State-Trait Anxiety Inventory-5; TAI-5 = Test Anxiety Inventory-5.

N = 311.

Cronbach α values reflect internal consistency for each total scale. Clustering of responses suggests potential ceiling effects, indicating that the current version may have limited ability to distinguish between neurophobic and non-neurophobic individuals.

Pretesting with medical students (n = 20) ensured content relevance and clarity. Feedback informed item refinement, and nonrelevant questions identified by expert consensus were revised or removed. A pretest pilot sampling study was performed using a small sample size followed by a second pilot study with larger sample size using an ideal ratio of 10 respondents for each scale item (10:1 ratio).^48^ The aim was to have a sample size of approximately 200–300 for factor analysis.^49^

### Psychometric Analysis

#### Statistical Analysis

All analyses were conducted using Stata version 17 (Stata Corp LLC, College Station, TX, USA). Descriptive statistics were calculated for demographic and scale variables. Data included ordinal (Likert-type), categorical (e.g., sex, entry status), and interval variables (e.g., MCQE scores). Spearman rank correlations were used to examine associations among variables.^50,e1,e2^

#### Content Validity

Content validity was established by a panel of 8 expert reviewers to rate the relevance and clarity of each item.^e3^ An inductive (item generation grouping items from responses from individuals) and deductive methodology (item generation from literature review and previous scales) was used for item generation.^e3,e4^ Individual interviews were performed with experts in the field including neurologists, neuropsychologists, and academic staff. Content analysis of items was pooled using a deductive approach from 2 previously validated scales: NeuroQ and Schön Questionnaire.^19,e4,e5^ Evidence of content validity was assessed by expert analysis using the Delphi method. Items were accepted, rejected, or modified based on majority opinion. Two iterative rounds were conducted. Items not meeting consensus were revised and reevaluated until ≥75% agreement was achieved.^e6^

#### Reliability and Item Analysis

Internal consistency was evaluated using Cronbach α and McDonald ω, with α ≥ 0.70 indicating acceptable and α ≥ 0.80 good reliability.^e7,e8^ Item discrimination and difficulty parameters were examined using Item Response Theory (IRT) under a graded response model (GRM). Model comparison was conducted using the Akaike Information Criterion (AIC) and Bayesian Information Criterion (BIC).

#### Construct Validity

Construct validity was examined in sequential stages. Exploratory factor analysis (EFA) identified underlying dimensions, with factors retained if eigenvalues exceeded 1.0, scree plot inflection was supported, and item loadings were ≥0.30.^e9,e10^ Confirmatory Factor Analysis (CFA) tested a 2-factor correlated model representing affective-attitudinal and self-efficacy domains. Model adequacy was evaluated using multiple indices: χ^2^/df ≤ 3, Comparative Fit Index (CFI) ≥0.90, Tucker-Lewis Index (TLI) ≥0.90, Root Mean Square Error of Approximation (RMSEA) ≤0.08, and Standardized Root Mean Square Residual (SRMR) ≤0.08.^e11^ Structural Equation Modeling (SEM) assessed a higher-order latent construct of overall neurophobia, integrating both domains within a unified measurement model.

#### Convergent, Discriminant, and Criterion Validity

Convergent validity was supported by item-total correlations ≥0.40 and significant correlations with related constructs (TAI-5, STAIT-5). Discriminant validity was indicated by weak correlations (<0.30) with unrelated measures. Criterion validity was evaluated through correlations between NCM scores and MCQE performance, whereas predictive validity was tested using linear and logistic regression models assessing whether NCM, TAI-5, and STAIT-5 scores predicted academic performance.^40,e12,e13^

#### Determining a Cutoff Score

The cutoff for high neurophobia was determined using receiver operating characteristic (ROC) analysis, with area under the ROC curve (AUC) calculated via the DeLong method to assess diagnostic performance.^e14^ As there is no current gold standard for a neurophobia psychometric instrument, the reference variable for “neurophobic” was classified using cutoff point sourced from the validated scale NeuroQ.^17^ The cutoff point was selected at the value maximizing both sensitivity and specificity, using previously established NeuroQ thresholds as an external comparator. The Schön Questionnaire was not used as a comparator due to its unvalidated thresholds.

## Results

### Participant Characteristics

Participants were recruited from final year medicine in 2022–2023 and 2023–2024 (Table 1). Of the 698 students invited, 311 (year 1 n = 169) and (year 2 n = 142) completed questionnaires. The response rate was 44.56%. The mean age of students was 24 years (SD = 3 years). A majority of students were female (n = 198, 63.7%), and 70.4% (n = 219) were undergraduate entrants.

### Descriptive Statistics

Item-level descriptive statistics and internal consistency for the NCM, TAI-5, and STAIT-5 are presented in Table 2. Across NCM items, means ranged from 2.50 to 3.81 (SD = 0.72–1.14), indicating adequate variability and no evidence of floor or ceiling effects. The frequency and percentage of responses to individual NCM items are reported in eTable 1. TAI-5 and STAIT-5 individual item response frequencies are reported in eTable 2.

### Receiver Operating Curve (ROC) Analysis

ROC curve analysis suggested that the NCM had strong discriminatory ability in classifying respondents as neurophobic or non-neurophobic (eFigure 1). The AUC was 0.9568 (standard error = 0.0095, 95% CI 0.9296–0.9776), a value generally interpreted as indicating excellent diagnostic accuracy.^e14-e16^ A cutoff score of ≥29 was identified as the point that balanced sensitivity (95.2%) and specificity (80.0%), with an overall correct classification rate of 88.1%. As the comparator was based on NeuroQ thresholds, which share some item overlap with the NCM, these results may be influenced by dependency between measures and should, therefore, be interpreted with appropriate caution.

Distribution assessment (eFigure 2) revealed a unimodal score distribution, with responses clustering around moderate-to-high NCM values and moderate overlap between students classified as neurophobic and non-neurophobic.

#### Neurophobic Students

The frequency of neurophobic students was calculated using the cutoff score of 29 on the NCM. Most students were neurophobic (n = 187, 60%), and most of them (subgroup n = 187) were female (69%) and undergraduate entry (65%).

### Reliability

#### Internal Consistency

The NCM demonstrated good internal consistency, with Cronbach α = 0.80 and McDonald ω = 0.81, both exceeding recommended thresholds.^e7,e8^ Comparable results were observed across subgroups (eTable 3).Corrected item-total correlations for NCM items ranged from 0.35 to 0.66, consistent with acceptable homogeneity and absence of redundancy.

### Validity

#### Content Validity

Using an inductive approach, item generation began with qualitative feedback from 20 medical students, yielding 11 preliminary items. Content validity was established through a 2-round Delphi process with 8 experts in neurology, neuropsychology, and medical education. Items with ≥75% agreement were retained; others were revised or removed. Three items were refined after Round 1, and full consensus was reached in Round 2, producing a final nine-item scale capturing perceived difficulty and confidence in neurology.

#### Construct Validity: EFA

Sampling adequacy for factor analysis was acceptable (Kaiser-Meyer-Olkin = 0.812). Bartlett test of sphericity was significant, χ^2^(36) = 745.21, *p* < 0.001, confirming the suitability of the correlation matrix for factor analysis.

**Figure 2:**
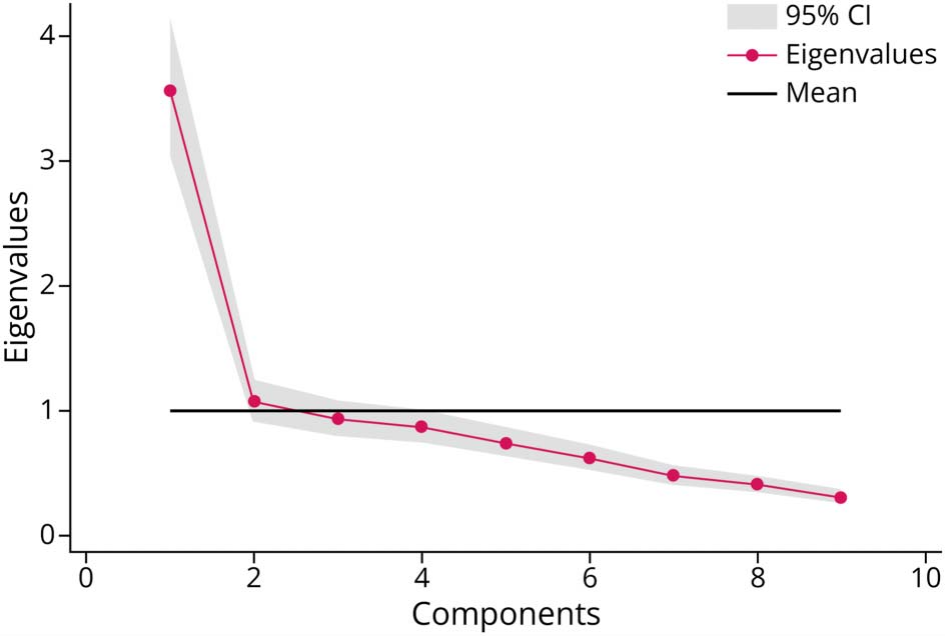
Scree Plot of Eigenvalues From Exploratory Factor Analysis of the 9-Item Neurophobia-Combined Measure (NCM) The plot displays eigenvalues on the y-axis and factor number on the x-axis. The “elbow” at the second factor, along with eigenvalues >1, supports a 2-factor solution. Factor 1 explained the largest proportion of variance (eigenvalue = 2.94), followed by Factor 2 (eigenvalue = 0.46). Parallel analysis results converged with the scree test in suggesting retention of 2 factors.

Principal axis factoring was conducted on the 9 items. In the unrotated solution, the first factor had an eigenvalue of 2.94, whereas subsequent factors had eigenvalues below 1.0 (eTable 4). Inspection of the FA-based scree plot (Figure 2) indicated an inflection after the second factor, supporting a 2-factor solution.

After varimax rotation, the 2-factor structure was interpretable as follows: Factor 1 (Perceived Difficulty/Complexity): items relating to the perception of neurology as difficult, complicated, or challenging to apply (e.g., SCHON3, NQ1, NQ3, NQ5). Factor 2 (Confidence/Interest): items reflecting self-rated confidence, knowledge, interest, and preparedness for neurology (e.g., SCHON1, SCHON2, SCHON4, NQ2, NQ4). Together, the 2 factors accounted for 51.4% of the variance. Rotated factor loadings and uniqueness values are presented in eTable 5.

#### Construct Validity: CFA

**Figure 3:**
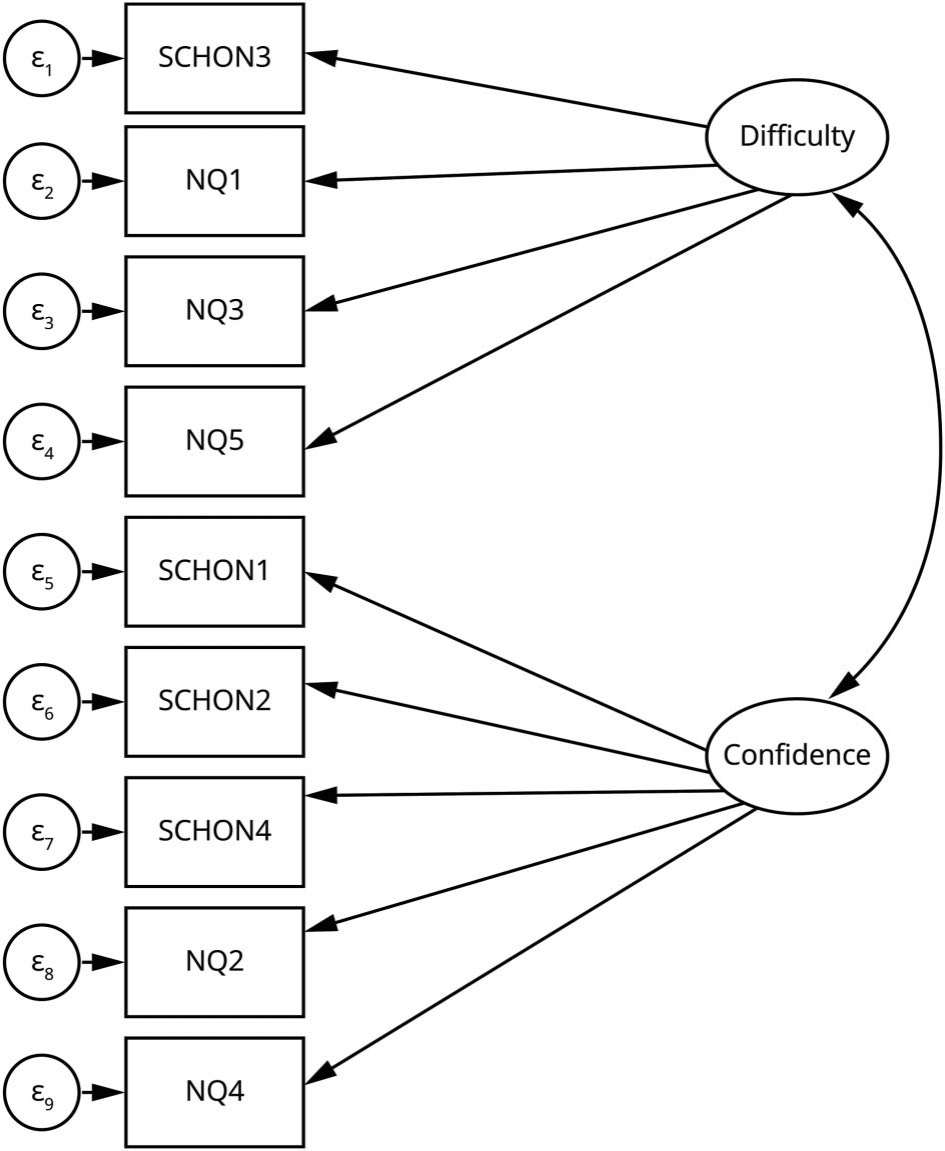
Structural Equation Model Demonstrating Relationships Between Perceived Neurology Difficulty (Difficulty) and Confidence in Neurological Knowledge and Clinical Skills (Confidence) Observed variables are represented by rectangles, latent variables by ellipses, and measurement errors by circles (ε_1_–ε_9_). Figure 3 depicts the structural equation model tested using the original coding of items (SCHON1–4, NQ1–5). These map directly onto the finalized items NCM1–9 in the Neurophobia-Combined Measure (NCM).

CFA was used to test the 2-factor solution. The correlated 2-factor model demonstrated acceptable fit: χ^2^(26) = 75.77, *p* < 0.001, RMSEA = 0.079, 90% CI [0.058, 0.099], CFI = 0.93, TLI = 0.90, and SRMR = 0.053. Standardized factor loadings ranged from 0.36 to 0.85, all significant at *p* < 0.001 (eTable 6). The correlation between the 2 latent factors was *r* = 0.69, 95% CI (0.60–0.79), indicating related but distinct constructs. A structural equation model is demonstrated in Figure 3.

For comparison, a unidimensional model was also tested and showed poorer fit: χ^2^ (27) = 148.27, *p* < 0.001, RMSEA = 0.120, 95% CI (0.102–0.140), CFI = 0.83, TLI = 0.78, and SRMR = 0.067. Full model fit indices are reported in eTable 7.

#### Convergent and Discriminant Validity

The average interitem correlation for the 9 NCM items was 0.31, within the recommended range of 0.15–0.50.^e17,e18^ By contrast, the STAIT-5 and TAI-5 had interitem correlations >0.50, suggesting stronger redundancy within those scales in this sample.

#### Criterion Validity

Criterion validity analyses showed statistically significant but small positive correlations between the NCM and both the Trait Anxiety Inventory (STAIT-5; ρ = 0.16, *p* = 0.004) and the Test Anxiety Inventory (TAI-5; ρ = 0.11, *p* = 0.047). Negative correlations were observed between the NCM and MCQE performance (ρ = −0.12, *p* = 0.041), and between TAI-5 and MCQE scores (ρ = −0.22, *p* < 0.001). The association between STAIT-5 and MCQE was small and nonsignificant (ρ = −0.09, *p* = 0.126). These findings provide evidence of criterion validity, while indicating that the associations were modest in magnitude,^e19^ which are presented in eTable 8 and Table 3.

**Table 3:** Fit Indices for Competing Confirmatory Factor Analysis Models

Model	χ^2^ (df)	RMSEA (95% CI)	CFI	TLI	SRMR	AIC	BIC
One-factor model	148.27 (27)^a^	0.120 (0.102–0.140)	0.83	0.78	0.067	6,770.18	6,871.15
Two-factor model	75.77 (26)^a^	0.079 (0.058–0.099)	0.93	0.90	0.053	6,699.68	6,804.39

Abbreviations: AIC = Akaike Information Criterion; BIC = Bayesian Information Criterion; CFI = Comparative Fit Index; RMSEA = Root Mean Square Error of Approximation; SRMR = Standardized Root Mean Square Residual; TLI = Tucker-Lewis Index.

N = 311.

a *p* < 0.001.

#### Predictive Validity

A multiple linear regression was conducted to examine whether neurophobia, test anxiety, state anxiety, sex, and academic entry status predicted neurology knowledge performance, as measured by MCQE scores. The overall model was statistically significant, *F* (5, 305) = 5.32, *p* < 0.001, and explained approximately 8.0% of the variance in MCQE scores, *R*^2^ = 0.08, adjusted *R*^2^ = 0.07.

Greater neurophobia was associated with lower MCQE performance, *B* = −0.07, SE = 0.03, β = −0.12, *t* = −2.08, *p* = 0.039, and 95% CI (−0.14 to −0.00). Similarly, higher test anxiety scores predicted lower knowledge performance, *B* = −0.17, SE = 0.05, β = −0.23, *t* = −3.40, *p* = 0.001, and 95% CI (−0.27 to −0.07). Academic entry status was also a significant predictor, with graduate-entry students performing better than undergraduate-entry students, *B* = 0.91, SE = 0.36, β = 0.14, *t* = 2.50, *p* = 0.013, and 95% CI (0.19–1.63).

#### IRT

GRMs were estimated for the Schön (4 items), NeuroQ (5 items), and combined NCM (9 items) scales (N = 311). Item parameters are shown in eTable 9. All discrimination estimates were statistically significant (*p* < 0.001) and ranged from a = 0.77 to 2.66 (95% CIs [0.50–3.33]), indicating moderate to very high discrimination according to established benchmarks (Baker, 2001). Thresholds were ordered monotonically for all items, with values spanning b = −6.03 to 4.15, demonstrating appropriate category functioning.

Model fit indices are presented in Table 4. The separate NeuroQ (−2LL = 3,585.76, AIC = 3,635.76, BIC = 3,729.25) and Schön models (−2LL = 2,999.92, AIC = 3,039.92, BIC = 3,114.72) showed better relative fit than the combined NCM model (−2LL = 6,457.44, AIC = 6,547.44, BIC = 6,715.73). Thus, although all items demonstrated acceptable psychometric functioning, comparisons indicate that the 2 scales are better modeled separately rather than as a single unidimensional measure. To evaluate the stability of item performance across models, a supplementary table summarizing discrimination parameters with 95% CIs for all items in the NeuroQ, Schön, and combined NCM models is provided (eTable 10).

**Table 4:** Model Fit Indices for Graded Response Models (n = 311)

Model	Items	−2LL	AIC	BIC
NeuroQ only	5	3,585.76	3,635.76	3,729.25
Schön only	4	2,999.92	3,039.92	3,114.72
Combined NCM	9	6,457.44	6,547.44	6,715.73

Abbreviation: AIC = Akaike Information Criterion; BIC = Bayesian Information Criterion; GRM = graded response model; NCM = neuro-combined measure.

Lower AIC/BIC values indicate better relative model fit.

## Discussion

This study developed and evaluated the NCM, a concise multidimensional tool designed to assess neurophobia among medical students. The NCM demonstrated good internal consistency, a 2-factor structure and satisfactory discriminatory ability to classify students as neurophobic. By integrating cognitive and affective domains from the Schön Questionnaire and the NeuroQ, the NCM addresses previous limitations in conceptual scope and psychometric consistency,^17,19,e20^ offering a more comprehensive and reliable assessment framework.

Criterion validity analyses indicated that higher neurophobia levels were associated with increased test anxiety and slightly lower performance on the MCQE, consistent with previous educational findings.^e19^ Although effect sizes were modest, these results support construct validity while suggesting that neurophobia interacts with broader emotional and contextual factors influencing learning.

IRT results were consistent with the 2-factor CFA model, indicating that the 2 scales collectively represent distinct but related dimensions of neurophobia. Separate models for each item demonstrated better fit than a single combined model, supporting the interpretation of 2 related constructs rather than 1 unidimensional scale. Factor analyses confirmed a 2-factor model encompassing perceived difficulty and confidence, aligning with multidimensional models previously hypothesized.^e10,e11^

Prevalence estimates were comparable to those reported in recent NeuroQ–based research, which has ranged from approximately 25% to 60% among medical student cohorts.^5,6,13,18,e21,e22^ Although some studies have observed modest declines in neurophobia following curriculum reforms and earlier clinical integration,^e23,e24^ rates remain consistently high. These differences may reflect variation in measurement sensitivity, as the NeuroQ's binary classification system tends to identify a broader proportion of students as neurophobic due to its emphasis on perceived difficulty rather than clinical competence.

The NCM's inclusion of knowledge, confidence, and perception domains may improve construct coverage, although further validation across settings is required. Academic anxiety correlated positively with neurophobia, consistent with evidence that anxiety reduces self-efficacy and engagement.^e25^ Female and undergraduate-entry students demonstrated higher neurophobia and test anxiety, highlighting potential targets for early educational interventions.^e26^ Undergraduate-entry students may encounter neurology earlier, with less clinical context, which could explain greater perceived difficulty and lower confidence. These findings underscore the importance of early, supportive teaching approaches and longitudinal research to clarify when and how neurophobia develops across training pathways.

Overall, the NCM provides an empirically supported framework for assessing neurophobia that advances measurement precision and educational applicability. Future work should validate the NCM across institutions, examine predictive risk factors, and explore its relationships with academic anxiety, self-efficacy, and clinical performance through longitudinal designs.

The NCM's discriminative validity may be limited, as item development occurred at a single center and score clustering reduced differentiation between neurophobic and non-neurophobic learners. Individuals who gave input into item generation for the NCM were from also from a single center. ROC-derived cutoffs, based on NeuroQ thresholds, may be sample-specific and upwardly biased because of item overlap. Future work should validate the NCM in diverse populations using independent outcomes and argument-based validity approaches to strengthen its psychometric evidence. Neurophobia may develop early, often during initial neuroanatomy teaching or first clinical exposures, and can persist if unaddressed.^35,e27^ As this study sampled only final-year students, it reflects attitudes at graduation but not their evolution throughout training. Limiting assessment to those completing the full curriculum may underestimate the influence of early educational experiences.

The NCM offers a reliable, multidimensional approach to assessing neurophobia in medical education. This study highlights the interaction between academic anxiety, performance, and perceptions of neurology. Although preliminary validation supports its utility, further refinement and broader validation are required. The NCM has the potential to guide targeted educational interventions and inform curriculum design to reduce neurophobia and enhance neurology learning outcomes.

## Glossary

AIU: Akaike's information criterion
AUC: area under the ROC curve
BIU: Bayesian information criterion
CFA: Confirmatory Factor Analysis
CFI: Comparative Fit Index
EFA: Exploratory factor analysis
GRM: Graded response model
IRT: Item Response Theory
MCQE: Multiple Choice Questionnaire Examination
NCM: Neurophobia-Combined Measure
RCSI: Royal College of Surgeons in Ireland
RMSEA: Root Mean Square Error of Approximation
ROC: Receiver operating characteristic
SRMR: Standardized Root Mean Square Residual
STAIT-5: State-Trait Anxiety Inventory
TAI-5: Test Anxiety Inventory
TLI: Tucker-Lewis Index
